# The response of coral skeletal nano structure and hardness to ocean acidification conditions

**DOI:** 10.1098/rsos.230248

**Published:** 2023-08-02

**Authors:** Chao Dun Tan, Georg Hähner, Susan Fitzer, Catherine Cole, Adrian A. Finch, Chris Hintz, Ken Hintz, Nicola Allison

**Affiliations:** ^1^ EaStCHEM School of Chemistry, University of St. Andrews, St. Andrews KY16 9TS, UK; ^2^ School of Earth and Environmental Sciences, University of St. Andrews, St. Andrews KY16 9TS, UK; ^3^ Institute of Aquaculture, Faculty of Natural Sciences, University of Stirling, Stirling FK9 4LA, UK; ^4^ Department of Marine and Environmental Sciences, Savannah State University, Savannah, GA USA; ^5^ Department of Electrical and Computer Engineering, George Mason University, Fairfax, VA, USA

**Keywords:** CaCO_3_, mechanical properties, biomineralization, environmental change

## Abstract

Ocean acidification typically reduces coral calcification rates and can fundamentally alter skeletal morphology. We use atomic force microscopy (AFM) and microindentation to determine how seawater pCO_2_ affects skeletal structure and Vickers hardness in a *Porites lutea* coral. At 400 µatm, the skeletal fasciculi are composed of tightly packed bundles of acicular crystals composed of quadrilateral nanograins, approximately 80–300 nm in dimensions. We interpret high adhesion at the nanograin edges as an organic coating. At 750 µatm the crystals are less regular in width and orientation and composed of either smaller/more rounded nanograins than observed at 400 µatm or of larger areas with little variation in adhesion. Coral aragonite may form via ion-by-ion attachment to the existing skeleton or via conversion of amorphous calcium carbonate precursors. Changes in nanoparticle morphology could reflect variations in the sizes of nanoparticles produced by each crystallization pathway or in the contributions of each pathway to biomineralization. We observe no significant variation in Vickers hardness between skeletons cultured at different seawater pCO_2_. Either the nanograin size does not affect skeletal hardness or the effect is offset by other changes in the skeleton, e.g. increases in skeletal organic material as reported in previous studies.

## 1. Introduction

Coral reefs underpin some of the world's most diverse ecosystems and provide ecosystem services which support fisheries and tourism and offer wave and storm protection for inland communities [1]. The reef structure is comprised of the mineral skeletons of corals consolidated with the biominerals of other calcareous organisms and inorganic CaCO_3_ precipitates [2]. For reef structures to persist in the future, their production must equal or exceed their destruction by dissolution and erosion [3]. Anthropogenic increases in atmospheric CO_2_ are altering seawater chemistry and reducing ocean pH in a process termed ocean acidification [4]. Ocean acidification typically decreases coral calcification (the rate at which the skeleton is formed) [5], results in pronounced changes in skeletal structure [6,7] and reduces the resistance of the skeletal structure to bioerosion [8,9]. Understanding why these changes occur is important for predicting the future of reef environments.

The coral biomineralization process is not completely understood. Until recently, the skeleton was believed to form at an extracellular calcification site between the base of the coral tissue and the underlying skeleton and to proceed via the attachment of aqueous ions (${\mathrm{CO}}_{3}^{2-}$ and Ca^2+^) to the existing aragonite skeleton [10]. Recent research suggests that coral aragonite also forms through a second crystallization pathway, via intracellular formation of amorphous calcium carbonate (ACC) precursors in vesicles in the basal layer of coral cells, the calicoblastic epithelium [11,12]. The contents of the vesicles are released at the base of the cells and the ACC particles move through the extracellular calcification media and attach to the skeleton, converting to aragonite either during transit or after attachment [12]. ACCs can convert to crystalline CaCO_3_ via solid-state transformation [13] or dissolution/reprecipitation [14].

The aragonite coral skeleton is composed of two key skeletal features [15], early mineralization zones (EMZ, [16]) and fasciculi. EMZ are also called centres of calcification or rapid accretion deposits [17], occur at the centres of the skeletal units and are composed of sub-micron randomly oriented crystals [18]. Fasciculi, also called thickening deposits or fibres [17], are composed of larger acicular crystals which radiate out in bundles from the EMZs to make up the bulk of the coral skeleton [19]. It is unclear how the two crystallization pathways relate to skeletal structure. Atomic force microscopy (AFM) indicates that the acicular skeletal fibres are composed of nanograins with typical dimensions of approximately 20–100 nm, coated with alternative materials which have been interpreted as organic in origin [17,20,21]. The skeleton of recently living coral contains approximately 2.5% organic materials plus water by mass after the tissue has been removed by sodium hypochlorite treatment [20]. This coral organic matrix is believed to play a role in the control of skeletal formation [22] as biomolecules can influence the nucleation [23], growth [24] and material properties [25] of different CaCO_3_ polymorphs. Concentrations of both the skeletal organics and skeletal amino acids increase in corals cultured under high seawater pCO_2_ [6,24,26]. These changes may affect skeletal structure and material properties.

In this research, we use AFM to explore the skeletal structures of a single coral colony, split into multiple sub colonies and cultured over a range of seawater pCO_2_ to mimic the effects of past CO_2_ atmospheres and future ocean acidification. AFM can measure sample topography at the nm scale [27] and can track sample adhesion (stickiness), an indicator of sample composition [28]. We collect topographic images to record the skeletal structure and adhesion micrographs to identify changes in the chemical composition of the skeleton which we infer to reflect the distribution of the organic matrix in the mineral. We analyse both the EMZ and fasciculi of the corals at nm scale. We also use microindentation to test how seawater pCO_2_ influences the hardness of the fasciculi of the skeletons. The material properties, e.g. hardness, fracture toughness and stiffness of calcareous structures, can indicate their likely resilience to erosion and predator/wave action [27]. We compare crystal structures, organic distributions and skeletal hardness across pCO_2_ treatments to gain a better insight into the effects of ocean acidification on coral biomineralization.

## 2. Methods

### 2.1. Coral culturing

In this study we analysed a single genotype of a massive *Porites lobata* coral cultured at 25°C and at different seawater pCO_2_ (approx. 180, 400 and 750 µatm). These CO_2_ concentrations simulate the environment in the Last Glacial Maximum, the present day and the seawater pCO_2_ expected by 2100 [29]. The coral culturing has been described previously in detail [30,31]. In brief, the coral was imported as a large adult head (approx. 25 cm in diameter) removed from a larger colony on a reef. Massive *Porites* spp. corals typically extend their skeletons by approximately 5–20 mm year [32] suggesting that the coral was >10 years old at the time of import. The imported coral was sawn into three sub colonies (each approx 12 cm in diameter) to provide sub-colonies for each seawater pCO_2_ treatment. The corals were acclimated to altered seawater pCO_2_ over two months and then maintained at target pCO_2_ for six months before sacrifice. Five weeks prior to sacrifice the corals were stained with alizarin red to create a time marker in each skeleton.

After sacrifice the coral skeletons were immersed in 3–4% sodium hypochlorite for ≥24 h with intermittent agitation to remove gross organic contamination, then rinsed repeatedly in distilled water and dried. Skeletal strips were sawn along the maximum growth axes, subdivided and embedded in epoxy resin (Epofix, Struers Ltd) in 25 mm diameter moulds. The coral mounts thus formed were polished using silicon carbide papers (up to 4000 grade, lubricated with water) and 3 µm and 0.25 µm diamond suspensions.

The coral studied here is genotype 1 as reported in our previous work [7,24,30]. High seawater pCO_2_ (750 µatm) reduced the calyx surface area in this coral, i.e. decreasing the mean diameter of individual coral polyps [7], but did not significantly affect calcification rate [30] or mean trabeculae width [7]. Total skeletal amino acid concentration was positively correlated with culturing seawater pCO_2_ in this coral [24].

### 2.2. AFM

AFM measurements were performed in ambient conditions with a Bruker Dimension Icon system (Santa Barbara, CA, USA) in PeakForce Tapping mode using V-shaped cantilevers (Bruker ScanAsyst-Air) of nominal spring constant 0.4 N m^−1^, and a nominal tip radius of 2 nm. In PeakForceTapping mode the cantilever oscillates vertically above the sample but does not come into direct physical contact with it. The cantilever tip and sample interact intermittently when they are proximate resulting in force distance curves from which information on sample height and adhesion can be extracted.

Adhesion forces between the tip and the surface depend on the (microscopic) contact area and hence roughness as well as on the chemical composition of tip and surface. A change in adhesion force therefore indicates a change in the contact area and/or the chemistry of the surface since the chemistry of the tip does not change. In addition, the thin water film that is present on the sample and tip establishes a capillary between the two that contributes to the adhesion. Adhesion forces are extracted from force distance curves by determining the maximum and minimum adhesion force, i.e. the difference between the force when tip and surface are in contact, and when they are completely separated [33].

The peak force set point was 3–12 nN, and the scan rate 0.4 Hz. Force–distance curves in PeakForceTapping mode were recorded with a frequency of 1 kHz while scanning across the mount surface. Adhesion forces were extracted from the retraction part of the force distance curves [33]. The average room temperature throughout the duration of the experiments was 21.7 ± 0.4°C and the relative humidity was 34%.

**Figure 1.**
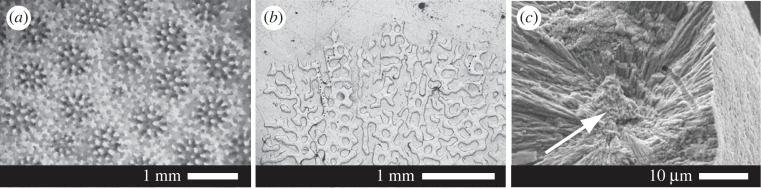
Morphology of *Porites lutea* corals. (*a*) Coral skeletal surface showing individual corallites photographed in reflected light and (*b*) polished block of section cut perpendicular to skeletal surface (along the maximum growth axis) photographed in reflected light. The skeleton consists of vertical rods (trabeculae) interconnected by horizontal rungs (synapticulae). (*c*) break across a single trabecula indicating EMZ (white arrow) and fasciculi, radiating out from the EMZ, imaged by scanning electron microscopy.

Atomic force topography and adhesion micrographs were collected concurrently across different skeletal structures in each sub-colony in 10 × 10 µm and 1 × 1 µm maps. Each micrograph was 512 × 512 pixels. *Porites* spp. corals produce perforate coral skeletons composed of interconnecting aragonitic rods (figure 1*a* and *b*). The vertical rods (oriented perpendicular to the growth surface of the skeleton) are called trabeculae while the horizontal rungs connecting the trabeculae are called synapticulae [15]. Each structure is composed of EMZs and fasciculi (figure 1*c*). We collected AF micrographs across EMZs and in the fasciculi, both adjacent to the EMZ (abbreviated to Fas_EMZ_) and adjacent to the outermost edge of the trabeculae (abbreviated to Fas_EoT_). We collected images across these areas in at least two different regions (deposited by different polyps) in each coral colony. All images were collected from the skeleton deposited in the five week period before the corals were sacrificed, i.e. after the alizarin red stain. It was not possible to identify accurately the skeletal structures at the surface in polished coral mounts by AFM. Blocks were briefly etched with 0.5% acetic acid for 30 s to create relief in the section surface. After etching, EMZs appeared as dark links or spots on the mount surface. AFM scans across the interface between the embedding resin and the coral aragonite indicating that etching removed about 300 nm of the surface carbonate.

### 2.3. Hardness

The mechanical hardness of calcareous structures can be estimated by measuring the resistance of the structure to irreversible deformation [27]. We measured the hardness of the coral skeletons using a Leco Vickers LM 248AT microindentation hardness tester equipped with a pyramid diamond indentor which was impressed into each sample using a mass of 100 g for a period of 10 s. Hardness varies depending on orientation in *Porites* spp. skeletons and is highest on samples prepared parallel to the growth surface of the coral and lower on samples which run perpendicular to the coral surface, i.e. along the main growth axis of the skeleton [34]. In the present study all measurements were made using the coral mounts used for AFM analysis, i.e. on samples that are cut perpendicular to the skeletal surface (figure 1*b*). Blocks were repolished after AFM to create a smooth surface which facilitated accurate measurement of indent dimensions. Analyses were made on the skeleton deposited in the final five weeks of the experiments (identified from the Alizarin Red S stain) and were sited on areas where the trabecula had reached a width of at least 100 µm. No analyses were made at the very top of the skeleton where the newly extended trabecula were narrow. Analyses were midway between the edge of each trabecula and the EMZs at the centre of the trabecula to avoid analysis of the sub-micron crystals which make up the EMZs. Analyses on the same trabecula were sited at least 100 µm apart. After analysis the mounts were photographed and the indent diagonal dimensions (D1/D2) were measured using ImageJ (National Institute of Health, USA) and used to calculate the indent area. Sample indents are shown in electronic supplementary material, figure S1. Most indents produced a diamond shaped scar. Occasionally the sample surface surrounding the scar appeared disturbed as if part of the sample surface had flaked away (electronic supplementary material, figure S1). These indents were excluded from the dataset. The Vickers hardness number (HV) was calculated from the ratio of the force applied by the indenter and the surface area of the final indent [27] as:

HV = (0.1891 × F)/(indent area, mm^2^); where *F* = applied force (*N*, in this case 0.9806)

HV was converted to Gpa by multiplying by 0.009807. A total of 25–26 microindentor measurements were made on each coral.

## 3. Results

### 3.1. Identification of skeletal structures by AFM

**Figure 2.**
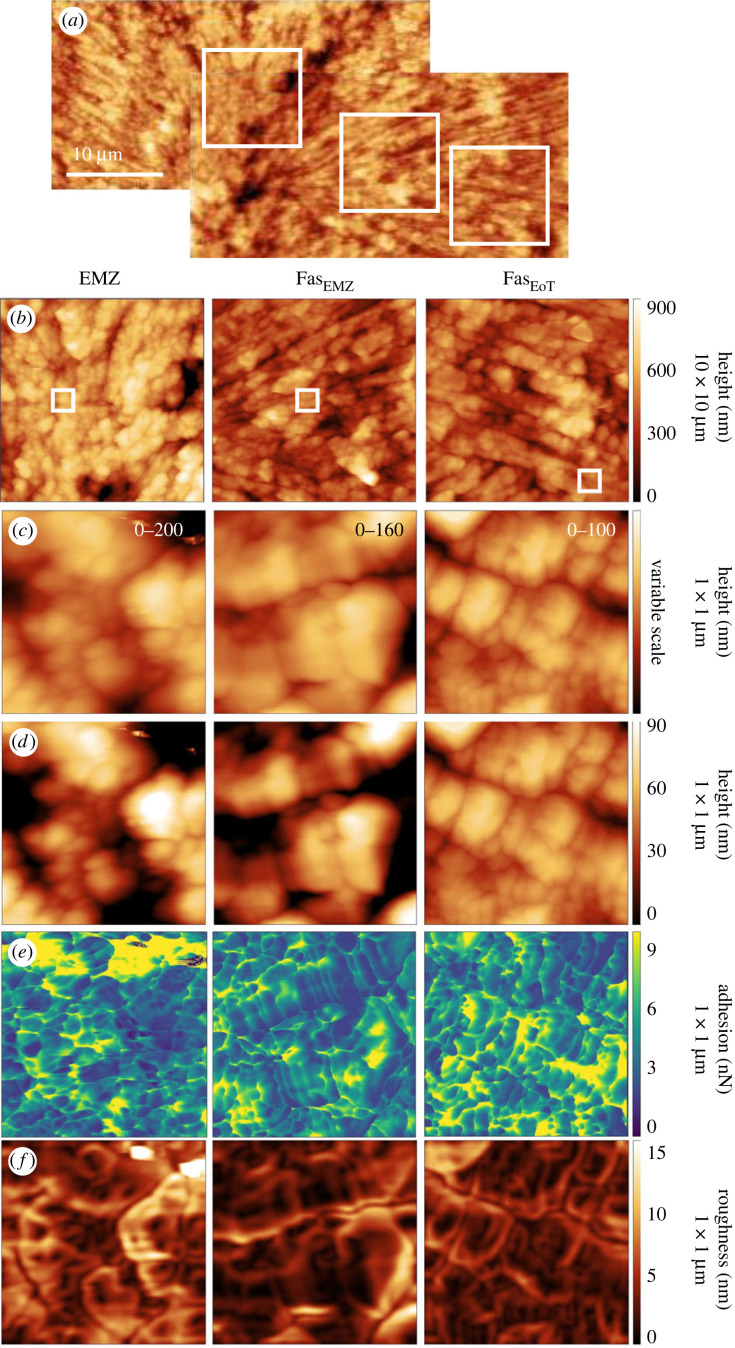
Atomic force micrographs of different skeletal features across a small area of the skeleton cultured at 400 µatm seawater pCO_2_. (*a*) A large area image was collected to orientate the sample. Variations in sample height were measured in selected areas at (*b*) 10 × 10 µm and at (*c*) and (*d*) 1 × 1 µm resolution. In (*c*) images are scaled from minimum to maximum height and the scale is superimposed on each image in white text. In (*d*) all images are scaled from 0–100 nm height. White boxes indicate the imaged areas. (*e*) Sample adhesion and (*f*) roughness are also shown for the 1 × 1 µm maps. Roughness is calculated as the standard deviation of sample height over 40 nm × 40 nm areas of the samples.

Atomic force micrographs confirm the typical coral skeletal structure (figure 2*a*). The EMZ are composed of small (<µm), randomly orientated crystals while the fasciculi (both adjacent to an EMZ and the edge of the trabecula) are composed of acicular crystals, typically about 1 µm across by tens of µm long (figure 2*b*). 1 × 1 µm micrographs detect subtle variations in sample height within the acicular crystals both across the approximately 1 µm width and along the length of each crystal (figure 2*c,d*). These divisions divide the acicular crystals into smaller entities with typical dimensions of approximately 80–300 nm. Adhesion varies several fold within each image (figure 2*e*). In the EMZs and fasciculi, higher adhesion values are observed at the interfaces between acicular crystals and also between entities within crystals. High adhesion values are also observed within entities in the fasciculi, frequently running parallel in direction to interfaces between entities (figure 2*f*). We identify the entities bounded by higher adhesion as nanograins within each crystal. High adhesion areas typically appear as thin lines (<10 nm across) but in some areas appear as larger patches up to 400 nm across (figure 2*e*, EMZ and Fas_EoT_ micrographs).

### 3.2. Adhesion in coral

**Figure 3.**
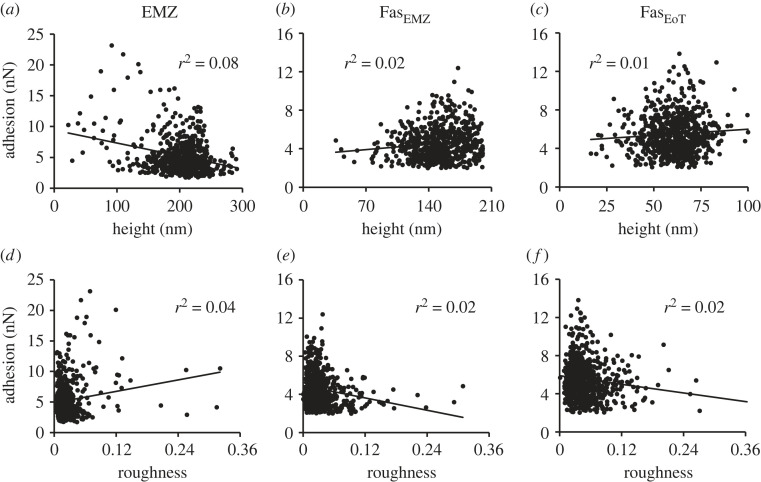
Relationships between sample adhesion and height and roughness for the micrographs in figure 2. Roughness is calculated as the standard deviation of sample height over a 40 nm × 40 nm area of the samples divided by the average sample height of the same area. Roughness scales from 0 (a completely smooth sample) to 1 (maximum roughness). Coefficients of determination are superimposed onto each graph.

To explore the influence of variations in sample height on adhesion across coral mounts, we plot adhesion as a function of sample height and sample roughness (figure 3, see electronic supplementary material, table S1 for values). The roughness of the sample is an indication of the change in surface topography local to each point in the 1 × 1 µm^2^ micrographs. Each micrograph is comprised of 512 × 512 pixels and roughness is calculated as the standard deviation of sample height for the 20 × 20 pixels (approx. 40 × 40 nm^2^) surrounding each pixel (figure 2*f*). We illustrate the relationships between adhesion and height or roughness for all the adhesion micrographs from figure 2 in figure 3. Coefficients of determination (*r*^2^) between the metrics are low (≤0.08) indicating that sample height and roughness has little effect on adhesion. Coefficients of determination (and *p* values) for all the corals studied in this paper are summarized in electronic supplementary material, table S2 and are always <0.09, and typically 0.02 for adhesion versus roughness and 0.04 for height versus roughness. Although *r*^2^ are small, any value ≥0.01 generates a significant *p* value (electronic supplementary material, table S2) due to the large number of points in each image.

### 3.3. Coral skeletal structure and seawater pCO_2_

**Figure 4.**
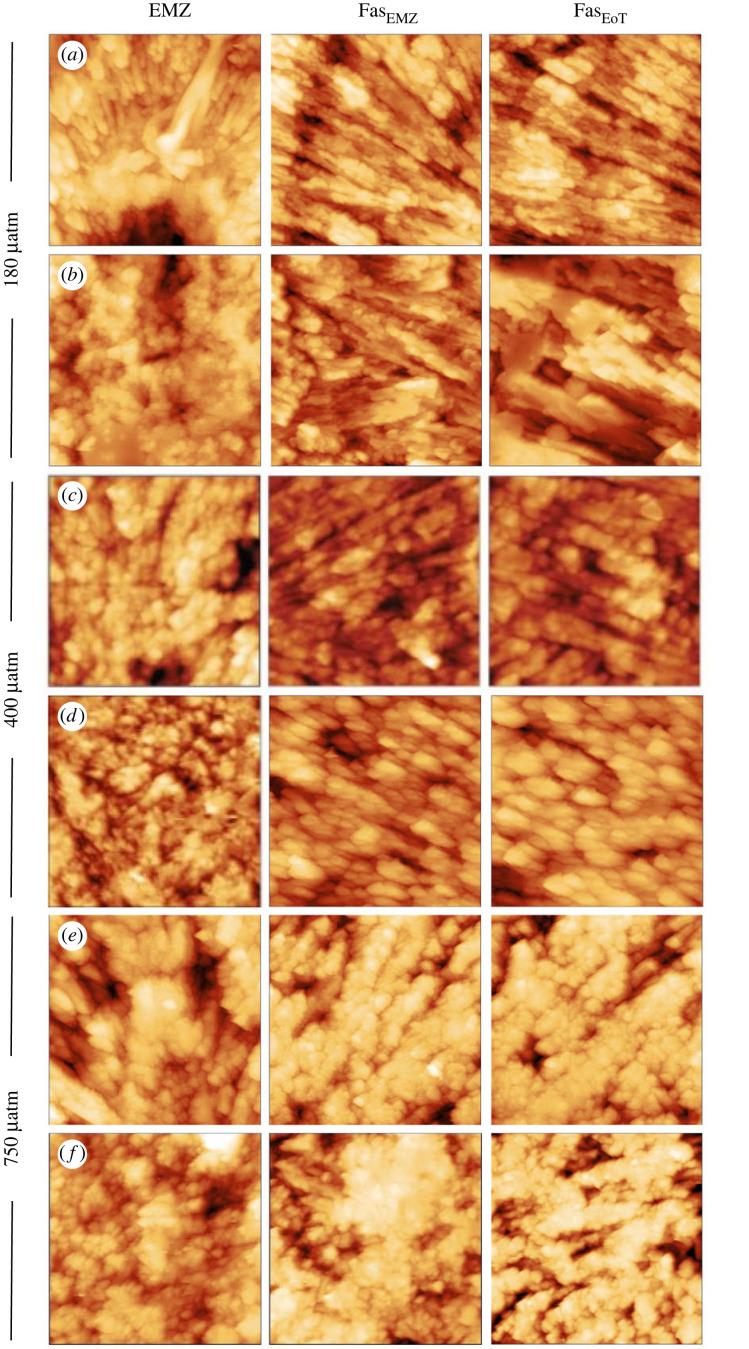
Atomic force sample height micrographs of skeletal features in corals cultured over a range of seawater pCO_2_. Each image is 10 × 10 µm.

**Figure 5.**
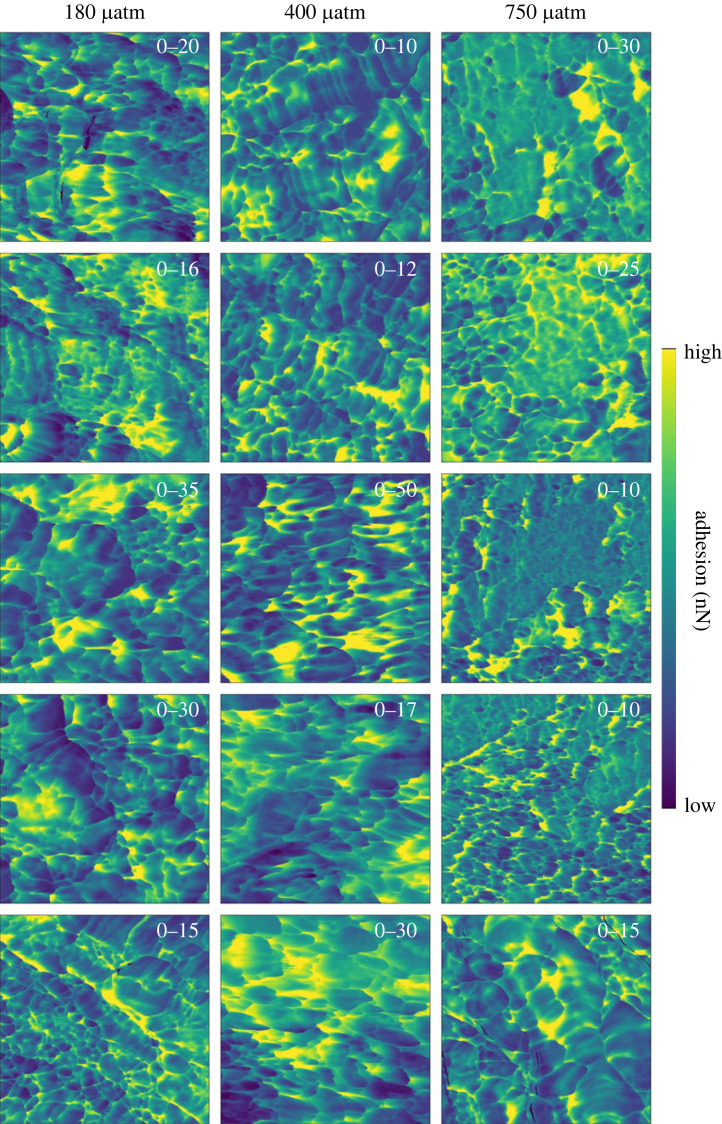
Atomic force adhesion micrographs of multiple 1 × 1 µm areas in fasciculi in each coral cultured at different seawater pCO_2_. Analyses from both FasEMZ and FasEOT are included. The adhesion scale is maximized for each image to highlight nanoparticle dimensions and the scale range is superimposed onto each image in white text.

**Figure 6.**
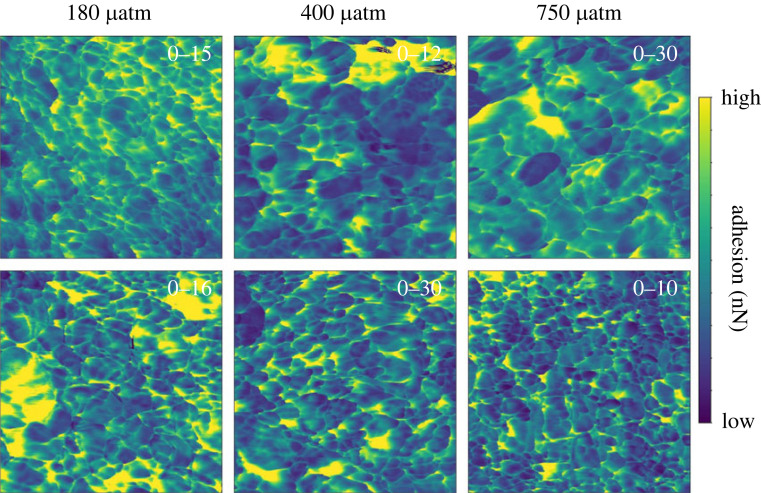
Atomic force adhesion micrographs of multiple 1 × 1 µm areas in EMZ in each coral cultured at different seawater pCO_2_. The adhesion scale is maximized for each image to highlight nanoparticle dimensions and the scale range is superimposed onto each image in white text.

AFM height images of the skeletons cultured at variable seawater pCO_2_ indicate that the fasciculi in the corals cultured at 180 and 400 µatm are composed of tightly packed bundles of acicular crystals with a width of approximately 1 µm and broadly straight edges (figure 4*a,b* and *c,d* respectively). In the coral cultured at high seawater pCO_2_ (750 µatm), the edges of the fasciculi crystals are irregular with a blobby appearance, the crystals are less regularly oriented and the entire structure appears more porous (figure 4*e,f*). The AFM adhesion micrographs indicate that at 400 µatm pCO_2_, the skeletal nanograins typically exhibit a quadrilateral to oval morphology with the particles typically elongated in one dimension (figure 5 central column) consistent with the orthorhombic symmetry of aragonite. The particles have dimensions of approximately 30–300 nm. At high seawater pCO_2_ (750 µatm) many nanograins are smaller (<25 nm) than in the coral cultured at 400 µatm and exhibit a more rounded morphology (figure 5 right column). However other areas of this coral exhibit little variation in adhesion over areas of approximately 200–800 nm (figure 5, right column). At low seawater pCO_2_ (180 µatm) the coral exhibits a mixture of elongate quadrilaterals (similar to at 400 µatm) and fine-grained nanoparticles (similar to at 750 µatm) but does not present the same large areas of low adhesion as observed in the high seawater pCO_2_ coral. Variations in the morphology of the EMZ crystals are not obvious between pCO_2_ treatments either in the sample height (figure 4) or in the sample adhesion (figure 6).

**Figure 7.**
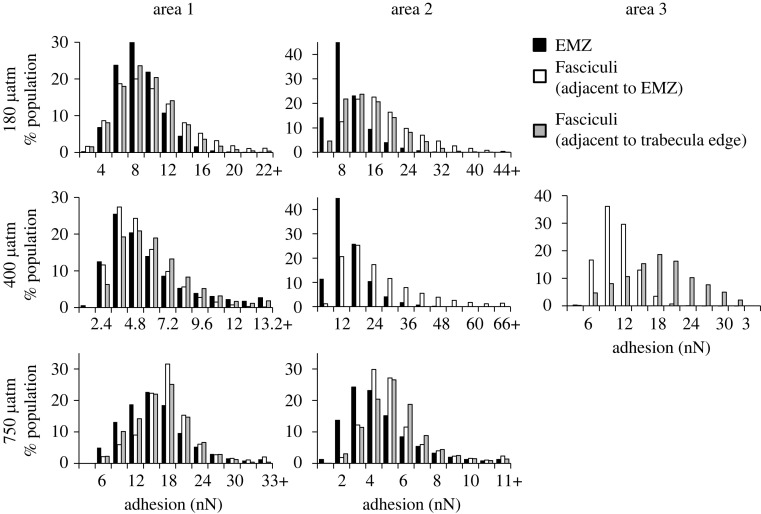
Population distributions of adhesion values (nN) from micrographs across different features in corals cultured over different pCO_2_. Adhesion can be influenced by relative humidity so features are only compared within an area when measured within a small time frame on the same day.

We compare the distribution of adhesion values between the different regions of each coral that were analysed on the same day (figure 7, see electronic supplementary material, table S3 for values). Sample adhesion can be affected by changes in relative humidity in the laboratory so we did not compare the adhesion between samples cultured at different seawater pCO_2_ which were analysed on different days. Adhesion values for each image (262144 pixels) were binned into 12 sequential classes (figure 7, electronic supplementary material, table S3) and the percentage of observations in each size class was calculated. Significant differences between distributions were identified using these percentages and the Kolmogorov–Smirnov test (table 1, *n* = 100). In three of the six regions where EMZs were analysed, the EMZs have a significantly different adhesion distribution compared to the other skeletal features and are offset to lower adhesion values (figure 7). In the other three areas no differences in adhesion distribution are observed between features. In a seventh area, the fasciculi adjacent to the EMZ has an adhesion distribution offset to significantly lower values than the fasciculi adjacent to the edge of the trabecula

**Table 1.** Summary of significant differences comparing adhesion distributions between different skeletal features i.e. EMZ, fasciculi adjacent to EMZ (termed Fas_EMZ_) and fasciculi adjacent to edge of trabeculae (termed Fas_EoT_) within each coral. Features were compared within a small area (within 100 µm) and up to three areas were analysed in each coral mount. All three different skeletal features were analysed in each area with the exceptions that only the EMZ and the fasciculi adjacent to EMZ were analysed in area 2 of the 400 µatm coral and only the fasciculi adjacent to EMZ and the fasciculi adjacent to the edge of the trabecula were analysed in area 3 of the 400 µatm coral. *p* values comparing distributions were calculated using the Kolmogorov–Smirnov test and values ≤ 0.05 are highlighted in bold.

	180 µatm	400 µatm	750 µatm
Area 1	EMZ = Fas_EMZ_ (*p* = 0.077)	EMZ = Fas_EMZ_ (*p* = 0.89)	EMZ = Fas_EMZ_ (*p* = 0.79)
	EMZ = Fas_EoT_ (*p* = 0.36)	EMZ = Fas_EoT_ (*p* = 0.14)	EMZ = Fas_EoT_ (*p* = 0.37)
	Fas_EMZ_ = Fas_EoT_ (*p* = 0.99)	Fas_EMZ_ = Fas_EoT_ (*p* = 0.37)	Fas_EMZ_ = Fas_EoT_ (*p* = 0.12)
Area 2	**EMZ** **≠** **Fas_EMZ_** **(*p* = 1.4 × 10^−11^)**	**EMZ** **≠** **Fas_EMZ_** **(*p* = 6.0 × 10^−6^)**	**EMZ ≠ Fas_EMZ_ (*p* = 2.1 × 10^−3^)**
	**EMZ** **≠** **Fas_EoT_** **(*p* = 1.8 × 10^−5^)**		**EMZ ≠ Fas_EoT_ (*p* = 0.35 × 10^−4^)**
	Fas_EMZ_ = Fas_EoT_ (*p* = 0.08)		Fas_EMZ_ = Fas_EoT_ (*p* = 0.67)
Area 3		**Fas_EMZ_** **≠** **Fas_EoT_** **(*p* = 4.0 × 10^−6^)**	

### 3.4. Skeletal hardness

**Figure 8.**
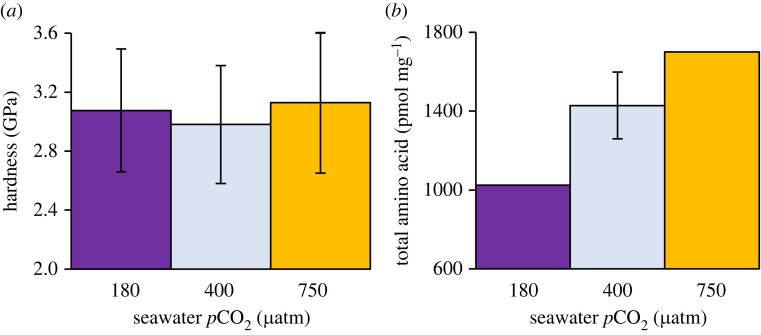
(*a*) Vickers hardness in the coral skeletons cultured at each seawater pCO_2_. 25–26 measurements were made in each coral. (*b*) Total amino acid content in each skeleton, reproduced from [24]. Error bars shows typical standard deviation of repeat analyses.

Between 25 and 26 hardness measurements were made on each skeleton (figure 8*a*). The typical diagonal dimensions of the indents were approximately 25 µm (electronic supplementary material, figure S1). Hardness values are normally distributed within each skeleton (Shapiro Wilk, *p* ≥ 0.97) and are not significantly different between the skeletons (one way ANOVA, *p* = 0.27).

## 4. Discussion

### 4.1. Adhesion as an indicator of biomolecules in coral skeletons

We observe large variations in adhesion in the AFM micrographs with higher values observed between skeletal nanograins. We considered whether changes in sample height or roughness could influence our adhesion measurements. The cantilever tip and sample interact intermittently when they are in proximity (termed the contact area). The total adhesion force between the tip and the sample reflects the chemical composition of the sample surface and the capillary force which is generated by interactions between the thin water films which occur on both the sample and cantilever tip under ambient conditions. Highly roughened surfaces (large variation in sample height) could potentially accumulate a deeper thickness of water or may display a higher adhesion if topography generates a higher contact area between the tip and sample. However, relationships between adhesion and either sample height or roughness are weak, i.e. <0.09 (figure 3, electronic supplementary material, table S2) and the full range of adhesion variation can be observed over areas of the sample which are almost flat (figure 2). Similarly large variations in sample height are not associated with variations in adhesion (figure 2). We conclude that variations in adhesion primarily reflect changes in the chemical composition of the sample.

**Figure 9.**
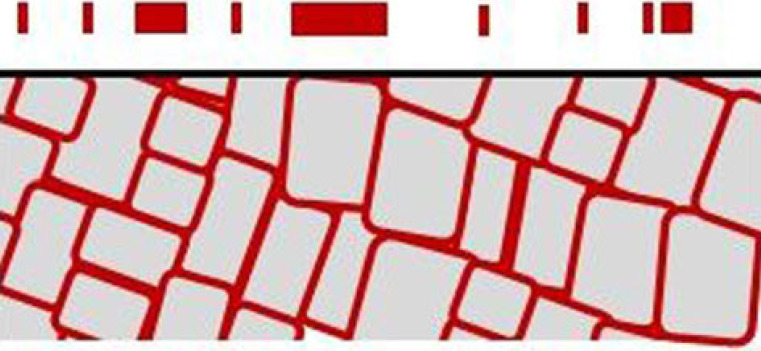
Schematic illustrating how organic coatings may appear in cross-section. The surface of the mount (black line) cuts across individual nanoparticles (in grey) so that organic coatings (red) typically appear as thin lines on the mount surface, illustrated by red bands hovering above the section. Occasionally the mount surface cuts between nanoparticles, exposing a broad width of organic coating at the surface.

Coral skeletons are composite materials of the mineral aragonite and an organic matrix (composed of proteins, polysaccharides and lipids [22]). Synthetic aragonites precipitated in the presence of aspartic acid contain higher concentrations of amino acids than aragonites precipitated with no biomolecules indicating that the amino acid is incorporated into the mineral [24]. Coral skeletal biomolecules are inferred to be present at the site of CaCO_3_ formation and are subsequently incorporated into the skeleton [22]. AFM phase contrast images of coral skeletons indicate the skeleton is composed of nanograins (typically tens of nm across) coated with alternative materials which have been interpreted as organic in origin [17,20,35] creating a composite material. In the present study we observe similar nanograins which exhibit higher adhesion values at their edges, reflecting changes in chemical composition. The most credible explanation for this observation is that the skeletal organic material is concentrated at the nanograin boundaries. The increases in adhesion typically appear as fine lines (<10 nm wide) surrounding the nanoparticles but occasionally appear as larger patches. The samples are cross-sections through the skeletons and the mount surface may cut through nanograins, exposing the grain boundaries in cross-section, or may cut between nanograins, exposing the boundary across a larger area (figure 9).

### 4.2. Adhesion and skeletal structures

**Figure 10.**
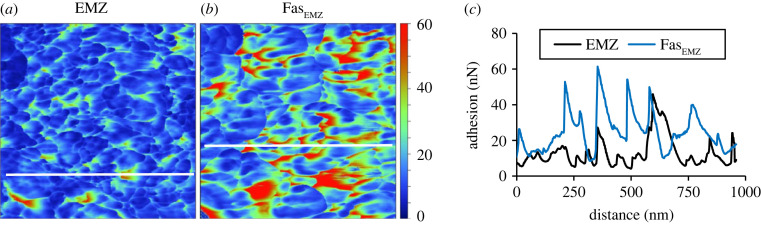
(*a*) and (*b*) Adhesion micrographs across the EMZ and FasEMZ of the coral cultured at 400 µatm (Area 2 in figure 7) shown at the same scale and (*c*) adhesion variations along the white lines in each image.

We find that the distribution of adhesion values measured in EMZs is frequently offset to lower values than observed in the fasciculi of the skeletons (figure 7). EMZs contain higher concentrations of organic matter than the surrounding fasciculi [18,36] and we anticipated that the EMZ areas would exhibit higher adhesion. We observe the opposite of this. The histograms compare adhesion values across the entire image, including both within and between nanograins (figure 7). To explore the variations in adhesion across nanograins we plot line transects across the adhesion micrographs (figure 10) for the EMZ and adjacent fasciculi in area 2 of the coral cultured at 400 µatm seawater pCO_2_, i.e. an area which exhibited a significant difference in adhesion distributions between EMZ and fasciculi. The lowest adhesion values occur within nanograins and are broadly comparable between the two skeletal regions (figure 10*c*). Highest values occur between nanograins and are usually higher in the fasciculi region than in the EMZ. The differences in the adhesion of the nanograin coatings in the two micrographs could reflect a change in organic composition between the two areas. For example, AFM adhesion varies significantly between calcite samples coated with different organic fatty acids [37]. Alternatively high adhesion in the fasciculi nanograin boundaries may indicate an increase in the thickness of the nanograin coating. Although we anticipate that EMZs have a higher organic content compared to fasciculi [36], the smaller nanograins in the EMZs have an increased surface area:volume ratio compared to the nanograins in the fasciculi. As an example, a 1 µm^3^ volume of solid composed of cubes of 20 nm in dimension has a total surface area that is 2.5 × greater than that of the same volume of solid composed of cubes of 50 nm in dimension. So a higher amount of organic material incorporated into the EMZ per unit volume as a thinner layer of organic material spread over a larger surface area of nanograins could explain the result observed here.

### 4.3. Skeletal structure and seawater pCO_2_

Our study detects changes in coral skeletal structure in response to ocean acidification, at both the µm and nm scale. In particular, at high seawater pCO_2_ the skeletal nanoparticles either become smaller and more rounded spheres or appear as larger areas with little variation in adhesion (figure 5). Aragonite in coral is inferred to form from conversion of amorphous calcium carbonate and from direct ion-by-ion attachment onto the existing aragonite skeleton [12]. It's not clear how these crystallization pathways relate to skeletal structure and if different processes are involved in the deposition of EMZs and fasciculi or in the deposition of the small nanograins versus the larger areas with little change in adhesion, as observed in the coral cultured at 750 µatm seawater pCO_2_. Our data suggest that changes in the crystallization process(es) occur in response to seawater pCO_2_. These could be changes in the contributions of different crystallization pathways, changes in the sizes of nanoparticles produced by each pathway or changes in the organic material incorporated in the skeletons.

Our data suggest that the skeleton deposited at 400 µatm is formed predominantly by a consistent process generating a repeating nanograin structure. The skeletons deposited at 750 µatm, and to some extent at 180 µatm, reflect more complicated nanograin structures which may indicate a greater diversity of biomineralization processes. Under ocean acidification conditions, the pH of the coral extracellular calcification media is reduced [31,38,39] while the organic and amino acid content of the skeleton is increased [6,24,26], (figure 8*b*). These changes could influence biomineralization by affecting the stability of any ACC phase e.g. during transit through the extracellular calcification media, as hypothesized by [12], or could reduce extracellular aragonite precipitation by ion-by-ion attachment. Intracellularly formed amorphous calcium carbonates and aragonites converted from them have been observed in the coral tissue, i.e. before they attach to the skeleton and have typical dimensions of approximately 500 to >1000 µm [11,12]. Spaces in skeletal aragonite, which are inferred to be infilled by ion-by-ion attachment, can be up to approximately 500 nm across [12]. These are both considerably larger than the nanoparticles sizes observed in the present study in the coral cultured at ambient pCO_2_ (400 µatm). Hydrated amorphous calcium carbonate particles shrink after dehydration [40] which could reduce particle size. Alternatively the amorphous calcium carbonate particles may crystallize themselves to create mosaics of nanograins. Further work is required to determine how crystallization pathways influence nanograin structure, e.g. via ion-by-ion aragonite precipitation or by amorphous calcium carbonate conversion to aragonite *in vitro*.

High seawater pCO_2_ also affects the coral skeletal structure at the micron scale resulting in crystals with more irregular dimensions and an apparent higher skeletal microporosity i.e. reflecting pores within individual trabeculae (figure 4). Increased microporosity has been reported previously in some corals growing at high seawater pCO_2_ [41,42] but was not observed in massive *Porites* spp. [42]. These changes indicate that corals do not simply produce less skeleton under ocean acidification but also alter the skeletal structure.

### 4.4. Skeletal hardness and seawater pCO_2_

Although we observe changes in the coral skeletal structure at the nano scale and in apparent skeletal porosity at the micron scale, these changes are not associated with a measurable change in structure hardness (figure 8*a*). We note that micro-indentation measures hardness at a scale of approximately 25 µm while AFM characterizes the skeletal structure at both the µm and nm scale. Hardness measurements may combine skeletal areas composed of small nanograins and areas of limited adhesion variation (as observed at 750 µatm seawater pCO_2_) but provide a broad comparison of the properties of the different skeletons. Either the micro- and nano-scale skeletal changes have no influence on the skeletal hardness or any changes are balanced via another mechanism. Inclusion of single amino acids increases the hardness of calcite [25] and nacre (the organic-aragonite composite coating the inside of mollusc shells) is many times more fracture resistant than inorganic aragonite [43]. Amino acid concentrations are higher in the coral cultured at 750 µatm seawater pCO_2_ in the present study (figure 8*b*) and this increase may act to improve the skeletal material properties. However the relationship between organic materials and coral skeleton properties is unclear. Coral skeletal proteins are highly organized and often co-located in the skeleton [44]. *Porites* spp. skeleton is typically harder in the organic rich aragonite formed at the skeleton surface compared to the older underlying material [45]. However microfracture stress and Young's modulus (stiffness) were significantly reduced in coral skeletons which contained high concentrations of organic material [45], suggesting that organic materials can also reduce mechanical resilience. Further work is required to determine how skeletal organics vary in response to seawater pCO_2_ and how such biomolecules influence material properties.

We use skeletal hardness as an indicator of the resilience of the structure to mechanical bioerosion as occurs when skeletons are bitten or scraped e.g. by parrotfish or urchins [46]. Hardness has indicated varying effects of ocean acidification in bivalve mollusc shells, i.e. ocean acidification increased the hardness of blue mussel shells [47] had no significant impact in clams [48] and juvenile eastern oysters [49] but reduced the hardness of Pacific oyster shells [50]. The ability of structures to resist physical wear and tear is a combination of hardness, fracture toughness and Young's modulus as well as the geometry of the physical contact, e.g. sharp or blunt [51]. It is preferable to test a range of properties to estimate the effects of ocean acidification on coral mechanical properties and our analysis should be viewed as a preliminary step. In addition, bioerosion also occurs by chemical attack. Boring sponges produce enzymes which directly dissolve and disintegrate the coral skeleton [52] and the respiratory CO_2_ of boring organisms, e.g. fungi and algae decreases the pH of intraskeletal porefluids [53] to promote carbonate dissolution. Assessing how changes in coral macrostructure [6,7], micro- and nano structure (this study) and organic content and composition [6,24,26] influence the response of skeletons to chemical attack is yet to be determined.

### 4.5. Implications for the response of corals to ocean acidification

In this study we observe changes in coral skeleton structure at the nanograin scale in response to ocean acidification. The colonies studied here were all sourced from a single *Porites lutea* parent colony which was imported as a single head and then divided to produce sub-colonies to culture in each seawater pCO_2_ treatment. Using a single genotype throughout ensures that variations between CO_2_ treatments do not reflect genetic variability but has the disadvantage that the effects of ocean acidification are tested on only one genotype. *Porites* spp. appear to be relatively resilient to ocean acidification and are found at naturally high pCO_2_ sites [54,55] when many other coral species are absent. There is broad variability between massive *Porites* spp. genotypes, both in terms of the calcification rates of individuals grown at present day seawater pCO_2_ [30] and in the response of corals to elevated pCO_2_ [7,30]. The individual coral selected for study here appeared relatively resilient to high seawater pCO_2_. The sub colony cultured at 750 µatm pCO_2_ had significantly smaller calices compared to the sub colonies cultured at 400 and 180 µatm [7] and higher skeletal amino acid concentrations (figure 8*b* after [24]). However calcification rates were broadly comparable between the seawater pCO_2_ treatments [30].

It is unclear if rapidly calcifying coral species or individuals are more susceptible or more resilient to ocean acidification than their slower growing analogues. The fastest calcifying massive *Porites* spp genotype in a recent study in our laboratory exhibited the most pronounced changes in skeletal morphology in response to ocean acidification [7]. Fast calcifier coral species also exhibited more pronounced reductions in calcification in response to increased seawater pCO_2_ than slower growing species [56]. Alternatively, slow growing coral species may be more vulnerable to amorphous calcium carbonate dissolution during biomineralization and therefore more susceptible to ocean acidification [57]. Further work is needed to understand the biomineralization process in coral and to explore the diversity of responses to ocean acidification between genotypes and species.

## Data Availability

All images are included in the manuscript. Supplementary data is included in electronic supplementary material, table S1–S4 [58].
